# Supplementary material for: Complexome profiling on the Chlamydomonas lpa2 mutant reveals insights into PSII biogenesis and new PSII associated proteins
Source: J Exp Bot. 2021 Aug 26;73(1):245–62. doi: 10.1093/jxb/erab390 (PMC8730698; doi:10.1093/jxb/erab390)
Supplement: erab390_suppl_Supplementary_Dataset_S1 [file erab390_suppl_supplementary_dataset_s1.zip › Supplemental Dataset 1 - Excel List and all profiles/plots/FKB16-8_Cre02.g095122.html]

Trivial name: FKB16-8  
  
Euclidean distance: 2286.15  
Mean Intensity (WT): 215.07  
Mean Intensity (Mut): 149.80  
Distance: 10.63  
  
MapMan: protein.folding.immunophilins (IMM).FKBPs;protein.folding  
  
p value of intensity sums Welch test: 0.3981
